# Evaluating and monitoring liver disease severity in glycogen storage disease type IX: Performance of novel and established clinical scores

**DOI:** 10.1016/j.gimo.2025.103455

**Published:** 2025-09-10

**Authors:** Anna Paschall, Rebecca L. Koch, Alisha M. Mavis, Leticia S. Flores, Rajan T. Gupta, William R. Jeck, Carolina Fischinger Moura de Souza, Bibiana Mello de Oliveira, Vikrant Sood, Seema Alam, Gilda Porta, Sheela Nampoothiri, Neerja Gupta, Jose Abdenur, Aruna Rikhi, Jessica Doxey, Andrew J. Muir, Priya S. Kishnani

**Affiliations:** 1 Division of Medical Genetics, Department of Pediatrics, Duke University Medical Center, Durham, NC; 2 Division of Gastroenterology, Hepatology and Nutrition, Department of Pediatrics, Duke University Medical Center, Durham, NC; 3 Division of Abdominal Imaging, Department of Radiology, Duke University Medical Center, Durham, NC; 4 Department of Pathology, Duke University Medical Center, Durham, NC; 5 Medical Genetics Service, Hospital de Clínicas de Porto Alegre, Brazil; 6 Department of Pediatric Hepatology and Liver Transplantation, Institute of Liver and Biliary Sciences, New Delhi, India; 7 Hepatology and Liver Transplant Unit, Menino Jesus Hospital, Brazil; 8 Department of Pediatric Genetics, Amrita Institute of Medical Sciences and Research Center, Cochin, India; 9 Division of Genetics, Department of Pediatrics, All India Institute of Medical Sciences, India; 10 Division of Metabolic Disorders, Department of Pediatrics, Children’s Hospital of Orange County, Orange, CA; 11 Duke Clinical Research Institute, Duke University Medical Center, Durham, NC; 12 Parexel International, Bonney Lake, WA; 13 Division of Gastroenterology, Department of Medicine, Duke University Medical Center, Durham, NC

**Keywords:** Glycogen storage disease type IX, Liver biopsy, Liver disease scoring, Liver disease severity, Liver fibrosis

## Abstract

**Purpose:**

Hepatic glycogen storage disease type IX (GSD IX) results from a deficiency of phosphorylase kinase. A noninvasive method of monitoring liver disease severity in this population is unavailable and liver biopsy remains the gold standard for classifying disease severity, particularly for liver fibrosis.

**Methods:**

We proposed 2 novel hepatic GSD IX-specific liver disease severity scoring systems: one that incorporated laboratory parameters (“novel lab-only score”) and one that incorporated laboratory, abdominal imaging, and growth parameters (“novel comprehensive score”). We compared their performance with established scoring systems used for other liver diseases and liver biopsy to determine concordance.

**Results:**

Liver disease severity was assessed in 56 pediatric participants with hepatic GSD IX. The novel lab-only and comprehensive scores correctly differentiated between mild and severe liver disease in 72.4% and 66.7% of participants, respectively. The Pediatric Non-Alcoholic Fatty Liver Disease Fibrosis Score demonstrated the highest accuracy in liver disease severity classification, correctly differentiating between mild and severe liver disease in 82.4% of participants.

**Conclusion:**

Hepatic GSD IX requires longitudinal monitoring and liver disease severity prediction scores may be useful clinical tools to noninvasively grade the severity of liver disease. Liver biopsy remains an important part of clinical management to understand the extent of liver disease.

## Introduction

Glycogen storage disease type IX (GSD IX) results from a deficiency of phosphorylase kinase (PhK), a key enzyme in glycogenolysis.^1^, ^2^, ^3^, ^4^, ^5^, ^6^ Although there are both muscular and hepatic subtypes of GSD IX, this study focuses on the hepatic subtypes of GSD IX. The clinical spectrum and severity of hepatic GSD IX varies widely by and within genetic subtypes: GSD IX α2 (OMIM 306000), β (OMIM 261750), and γ2 (OMIM 613027)—historically referred to as GSD IX types a, b, and c, respectively.^7^ Hepatic GSD IX may present early in life with mild to moderate hepatomegaly, growth retardation, fasting ketotic hypoglycemia, and elevated transaminases and plasma lipids.^1^, ^2^, ^3^, ^4^, ^5^, ^6^^,^^8^, ^9^, ^10^, ^11^, ^12^, ^13^ Individuals with severe GSD IX phenotypes can progress from hepatic fibrosis to cirrhosis, develop hepatic adenomas or hepatocellular carcinoma, or require liver transplantation.^1^^,^^10^^,^^11^^,^^13^

Liver biopsy currently remains the gold standard for determination of liver disease severity, particularly for liver fibrosis. Yet, liver biopsies are considered invasive, expensive, not readily repeatable, potentially unsafe in the setting of liver dysfunction, and only sample approximately 1/50,000th of the liver.^14^ Clinicians have used prediction scores to estimate liver disease severity in a number of liver pathologies (ie, Non-Alcoholic Fatty Liver Disease [NAFLD], hepatitis, and cirrhosis); however, no prediction score currently exists for hepatic GSD IX and other inborn errors of metabolism. Recent literature revealed that there is clinical variability in the severity of hepatic GSD IX,^7^ indicating that clinical prediction scores for hepatic GSDs are needed. Established scores for NAFLD, nonalcoholic steatohepatitis, and cirrhosis rely primarily on laboratory biomarkers and, with few exceptions, are only validated for use in an adult population.

Three scores have been proposed for use within the pediatric population: the Pediatric NAFLD Fibrosis Index, Pediatric NAFLD Fibrosis Score (PNFS), and the methodology proposed by Kulkarni et al.^15^, ^16^, ^17^, ^18^ Initial studies utilizing the proposed pediatric scores demonstrated good performance but either lack external validation or have demonstrated poor external validity.^15^^,^^19^, ^20^, ^21^ Use of these established scores for a hepatic GSD population is further limited because the studies explicitly excluded participants with metabolic disorders and demonstrated the best performance in detection of severe liver disease.^14^^,^^15^^,^^17^^,^^21^ Because most patients with GSD IX initially demonstrate mild to moderate liver disease and are more slowly progressive with variable rates of disease progression, these established scores may not accurately predict liver fibrosis severity or fail to capture the disease progression characteristic of hepatic GSDs.^22^ Because severe elevations or declines of transaminases may be present with acute liver inflammation or worsening liver fibrosis, cirrhosis, and liver failure, respectively, sole reliance on these biomarkers may lead to over- or underestimation of severity of liver disease in hepatic GSDs, especially for participants who retain liver synthetic function with simultaneous progression of liver fibrosis and a decrease in liver transaminases.^22^

Because no scoring systems exist for hepatic GSDs, we examined the established liver disease severity scores for other hepatic processes that incorporate key biomarkers. We additionally proposed 2 novel hepatic GSD IX-specific liver disease prediction scores, a “lab-only score” based on laboratory parameters, and a “comprehensive score,” which incorporated biomarkers, clinical growth parameters, and abdominal imaging data. We theorized that incorporation of imaging and growth parameters may improve performance of a clinical score in which severity of laboratory parameters may not accurately reflect the degree of liver disease. Performance of the novel and established scores were compared with the severity of liver disease on liver biopsy, where available. If successful, creation of a GSD IX-specific liver disease scoring system for predicting and monitoring progression of liver disease could guide disease management, identify patients at risk for worsening liver status, eliminate the need for multiple invasive biopsy procedures, and serve as an endpoint for future clinical trials.

## Materials And Methods

### Participant population

As part of an international collaboration, individuals ≤ 18 years old with a confirmed diagnosis of hepatic GSD IX were included for participation in this longitudinal, retrospective natural history study. Diagnosis of hepatic GSD IX was confirmed through genetic analysis by the presence of variant(s) in the PhK genes (*PHKA2* [HGNC:8926; NM_000292.3], *PHKB* [HGNC:8927; NM_000293.3], or *PHKG2* [HGNC:8931; NM_000294.3]).

### Data collection

All available paper and electronic records were reviewed for participants directly consented to the study. For participants included under the waiver of informed consent, a study-specific spreadsheet was provided to each collaborating institution who contributed deidentified participant information. Study data were entered into REDCap electronic data capture tools.^23^^,^^24^ Intermittent checks of REDCap records were performed throughout data entry.

### Clinical history

General demographic information was collected for all participants including sex, race, and ethnicity. Diagnostic information included age at diagnosis, genetic variants, and enzymology. Age at clinical visit and anthropomorphic parameters including height (cm), weight (kg), and *z*-score-adjusted growth parameters were documented. All available reports from abdominal imaging (ie, ultrasound [US], computed tomography [CT], and magnetic resonance imaging [MRI]) and liver biopsies performed as part of routine clinical care were recorded.

### Development of Novel GSD IX Liver Disease Severity Score System

A novel liver disease scoring system for use in pediatric populations was developed through collaboration with hepatologists and a radiologist with expertise in abdominal imaging (Table 1). Severity of liver disease at baseline and most recent visit was classified as absent/normal, mild (early-stage liver disease), moderate (fibrosis), or severe (cirrhosis) using the following parameters:

1. Laboratory investigations
2. Abdominal imaging
3. Growth measurements

**Table 1:** Components of the proposed Novel GSD IX Liver Disease Severity Scoring System in pediatric populations

Parameters	Laboratory Investigations CTCAE Grade	Abdominal Imaging Classification	Growth (*z*-Score)
	ALT, GGT, Bilirubin	US, CT, or MRI	Weight-For-Length, Weight-For-Age, Stature-For-Age, BMI-For-Age
Mild Score = 1	Grade 1 of ≥ 1 defined parameters	US: hepatomegaly, minimal increased echogenicity	−0.5 to 0 SD below the mean
		CT: hepatomegaly, minimal increased echogenicity, decreased attenuation of the liver, noted hepatic steatosis	
		MRI: hepatomegaly, signal drop-out on opposed imaging, noted hepatic steatosis	
Moderate Score = 2	Grade 2-3 of ≥ 1 defined parameters	US: increased echogenicity, heterogeneous/coarse echotexture, disruption of septal architecture	Between −0.5 to −1 SD below the mean
		CT or MRI: heterogeneous enhancement of the liver, mild nodularity, profound hepatic steatosis	
Severe Score = 3	Grade 4 of ≥ 1 defined parameters	US: shrunken, nodular contour	≥−1 SD below the mean
		CT or MRI: nodular or cirrhotic morphology	

The scoring system incorporated laboratory parameters, abdominal imaging, and growth parameters. Abdominal imaging classification criteria was developed by an experienced radiologist specialized in abdominal imaging. Participants were scored for severity in each parameter: normal (0), mild (1), moderate (2), and severe (3).

*ALT*, alanine aminotransferase; *CT*, computed tomography; *CTCAE*, Common Terminology for Adverse Events; *GGT*, gamma-glutamyl transferase; *GSD IX*, glycogen storage disease type IX; *MRI*, magnetic resonance imaging; *SD*, standard deviation; *US*, ultrasound.

Severity of elevations in laboratory investigations were graded using age-adjusted upper limits of normal in conjunction with the Common Terminology for Adverse Events version 4.0. Alanine aminotransferase (ALT) was used as a marker of necro-inflammatory activity, bilirubin as a marker of liver function, and gamma-glutamyl transferase (GGT) as a potential marker of advanced fibrosis.^15^ ALT was preferentially selected over AST (aspartate aminotransferase) because elevations in AST are less specific for the liver and can be skewed by myopathy.^25^ Moreover, GGT rather than ALP (alkaline phosphatase) was selected in the pediatric population because baseline ALP values may be falsely elevated because of bone growth.^26^ For abdominal imaging classification, each available written radiology report was reviewed and categorized by severity of findings. Imaging results with absence of liver abnormalities were classified as normal.

### Calculation of novel lab-only and comprehensive scores

The parameters measured in the Novel GSD IX Liver Disease Severity Score System were then used to calculate 2 scores:

1. Novel lab-only score, which only incorporated laboratory parameters.
2. Novel comprehensive score, which incorporated laboratory, imaging, and growth parameters. Participants were classified to the greatest severity they qualified for based on the laboratory, abdominal imaging, and growth parameters.

Calculation of the novel lab-only score allowed for comparison of performance of the novel comprehensive score with and without abdominal imaging data to account for participants that did not have abdominal imaging data available.

### Calculation of established severity scores

Classifications of liver disease severity utilizing the novel scoring system were compared with classifications calculated from 4 established liver disease scoring systems: PNFS, Kulkarni et al,^16^ AST to Platelet Ratio Index (APRI), and Fibrosis-4 Index (FIB-4). Although the APRI and FIB-4 scores have only been validated for use in an adult population, previous literature has demonstrated that these scores outperform or perform similarly to the pediatric scores in detecting fibrosis and differentiating between insignificant and significant fibrosis.^14^^,^^15^^,^^21^^,^^27^ The equations used for each score can be found with the original references.^15^^,^^16^^,^^28^, ^29^, ^30^ In addition to reporting summary statistics, each score’s previously defined cutoffs to divide each score into mild, moderate, and severe were utilized to more easily compare performance of the novel and established scores (Table 2). Lastly, the Pediatric End-Stage Liver Disease (PELD) scoring system is often used to prioritize a patient for liver transplantation.^28^ Classification of liver disease severity using the PELD scoring system was calculated and reported using summary statistics; use of the severity cutoffs was not possible because it was originally developed to estimate mortality risk. The Pediatric NAFLD Fibrosis Index was not utilized in this study because waist circumference information^17^ was not available for our cohort.

**Table 2:** Established liver disease clinical score severity classification

Liver Disease Scoring System	Equation	Predicted Liver Fibrosis Severity		
		Mild	Moderate	Severe/Cirrhosis
PNFS	$z=1.1+\left(0.34\;x\;\sqrt{ALT}\right)+\left(0.002\;x\;Alkaline\;Phosphatase\right)-\left(1.1\;x\;\log\left(Platelets\right)\right)-\left(0.02\;x\;GGT\right)$ $p=\left[\frac{e^{z}}{1+e^{z}}\right]x\;100$ where z= logistic regression and p= probability distribution	<13.2	13.2-24.7	>24.7
FIB-4	$\left(Age\;x\;AST)/(Platelet\;count\;\left(10^{9}\right)x\surd ALT\right)$	<1.45	1.45-3.25^a^	>3.25
Kulkarni et al^16^	Σ[BMI%x0.07,GGTx0.04,25−OHVitaminDx(−0.5),Plateletsx(−0.03)]	Scores lower than −6.13 predict presence of fibrosis		
APRI	$\frac{\left(\frac{AST}{upper\;limit\;of\;normal}\right)x100}{platelet\;count}$	<0.7	0.7-1	>1

The defined cutoff criteria for the Pediatric Non-Alcoholic Fatty Liver Disease Score (PNFS), Kulkarni et al,^16^ AST/Platelet Ratio Index (APRI), and Fibrosis-4 Index (Fib-4) scoring systems are listed. To more easily compare performance of these established scores with the novel scoring system, the cutoff criteria are classified by severity: mild, moderate, or severe.

a Using the Fib-4 system, a value from 1.45 to 3.25 is considered indeterminate.

### Histological classification and comparison

For participants with liver biopsy data, pathology reports for each specimen were collected. Whole slide images were unavailable from all collaborating institutions; therefore, pathology reports detailing microscopic findings were used in favor of selected portions of slides to minimize selection bias. Each report was analyzed by a collaborating pathologist (W.J.) with expertise in liver histopathology and graded using both the Ishak and Batts-Ludwig staging system. The Ishak stage was utilized because it divides findings into 7 categories, increasing the ability of the observer to categorize small differences in histology, and the Batts-Ludwig stage was reported for comparison because it represents conversion of the International Association for the Study of the Liver descriptions into a 4-category score.^31^ For clinical relevance and to aid comparison with the proposed novel comprehensive score, the Batts-Ludwig and Ishak stages were divided and defined as mild, moderate, or severe, as represented in Supplemental Table 1. With any discrepancies between the 2 staging systems for severity score, we deferred to the Ishak grade because of the improved differentiation provided by its 7 categories.

To compare performance between scores, we used the liver disease severity score determined by histology grade (gold standard) and compared it with liver disease severity scores calculated at the clinical visit closest to liver biopsy. Classification performance of Kulkarni et al^16^ methodology, APRI, or PELD scores were not explored because of a number of limitations: (1) the Kulkarni et al^16^ methodology only provides a single cutoff value for predicting absence or presence of fibrosis, not severity; (2) the APRI placed almost the entire GSD IX cohort in mild disease, regardless of genetic subtype; and (3) the PELD as stated earlier was designed for predicting transplant mortality and increase with sequelae of portal hypertension; given that portal hypertension was only present in a few individuals (*n* = 3, age range 3.3-13.5 years) in our cohort, all of our participants had very low scores of limited clinical importance.

### Statistical analysis

Statistical analysis was performed at Duke University using SAS (Version 9.4) and GraphPad Prism 9. The calculation of percentages was based on the number of participants with nonmissing measurements for that time point in the corresponding category. When comparing GSD IX subtypes, continuous variables were analyzed using the Kruskal-Wallis test. Categorical variables were compared using χ^2^ or Fishers exact test. Scoring variables were imputed based on age of observation. For the imputation rule, if a laboratory parameter was missing and a value was available for that parameter within the same chronological year of age as the visit in question, the value was imputed with the available value at the later clinical time point. Use of the imputation rule increased the number of participants for which a score could be calculated because laboratory parameters were often collected at multiple time points within a short time frame.

## Results

**Figure 1:**
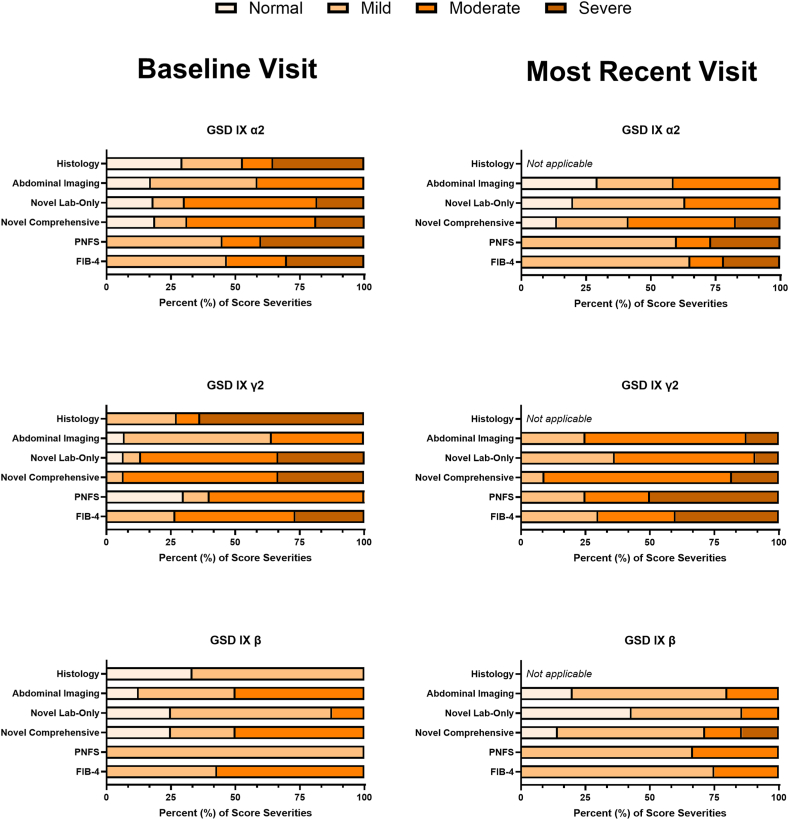
**Liver disease score severity comparisons in all pediatric participants with hepatic GSD IX at baseline and most recent visit.** Liver disease severity classifications at baseline and most recent visit (MRV) were separated by the GSD IX subtypes (α2, γ2, and β). The severity was assessed by histology, abdominal imaging, novel lab-only, novel comprehensive, Pediatric Non-Alcoholic Fatty Liver Disease Score (PNFS), and Fibrosis-4 Index (FIB-4) scoring systems. The number of participants included in each score is included in Table 3. GSD IX, glycogen storage disease type IX.

There were 57 pediatric participants (45 males and 12 females) with a genetically confirmed diagnosis of hepatic GSD IX included in the study (Supplemental Tables 2 and 3). Overall, 34 (59.6%) of participants had GSD IX α2, 15 (26.3%) had GSD IX γ2, and 8 (14.0%) had GSD IX β (Table 3). Figure 1 provides the severity classification across subtypes for novel and established liver disease scoring systems at baseline and most recent visit.

**Table 3:** Liver disease severity classifications in pediatric participants with hepatic GSD IX by genetic subtype at their baseline evaluation compared with their most recent visit

Parameter	GSD IX α2 *N* = 34		GSD IX γ2 *N* = 15		GSD IX β *N* = 8		All Participants *N* = 57	
	Baseline	Most Recent Visit	Baseline	Most Recent Visit	Baseline	Most Recent Visit	Baseline	Most Recent Visit
Histology								
*N*, Median age (25th-75th)	17, 3.1 (2.0-4.1)	N/A	11, 1.6 (1.0-4.0)	N/A	3, 2.0 (1.8-4.1)	N/A	31, 2.9 (1.6-4.1)	N/A
Normal (%)	5 (29.4%)	N/A	0 (0.0%)	N/A	1 (33.3%)	N/A	6 (19.4%)	N/A
Mild (%)	4 (23.5%)	N/A	3 (27.3%)	N/A	2 (66.7%)	N/A	9 (29.0%)	N/A
Moderate (%)	2 (11.8%)	N/A	1 (9.1%)	N/A	0 (0.0%)	N/A	3 (9.7%)	N/A
Severe (%)	6 (35.3%)	N/A	7 (63.6%)	N/A	0 (0.0%)	N/A	13 (41.9%)	N/A
Abdominal imaging								
*N*, Median age (25th-75th)	30, 3.4 (2.0-4.9)	17, 8.0 (6.1-9.9)	15, 1.6 (0.9-4.6)	8, 8.2 (2.2-9.9)	8, 5.0 (2.7-7.1)	5, 10.2 (8.1-13.0)	54, 3.2 (1.8-5.0)	30, 8.4 (6.1-10.3)
Normal (%)	5 (16.7%)	5 (29.4%)	1 (6.7%)	0 (0.0%)	1 (12.5%)	1 (20.0%)	7 (13.0%)	6 (20.0%)
Mild (%)	12 (40.0%)	5 (29.4%)	8 (53.3%)	2 (25.0%)	3 (37.5%)	3 (60.0%)	24 (44.4%)	10 (33.3%)
Moderate (%)	12 (40.0%)	7 (41.2%)	5 (33.3%)	5 (62.5%)	4 (50.0%)	1 (20.0%)	21 (38.9%)	13 (43.3%)
Severe (%)	0 (0.0%)	0 (0.0%)	0 (0.0%)	1 (12.5%)	0 (0.0%)	0 (0.0%)	0 (0.0%)	1 (3.3%)
ND (%)	1 (3.3%)	0 (0.0%)	1 (6.7%)	0 (0.0%)	0 (0.0%)	0 (0.0%)	2 (3.7%)	0 (0.0%)
Novel lab-only score								
*N*, Median age (25th-75th)	33, 2.7 (1.7-3.5)	30, 6.9 (5.0-8.6)	15, 1.4 (0.9-3.8)	11, 7.2 (5.6 -9.7)	8, 3.5 (0.5-5.3)	7, 10.1 (8.2-13.8)	56, 2.1 (1.1-4.3)	48, 7.5 (5.1-9.6)
Normal (%)	6 (18.2%)	6 (20.0%)	1 (6.7%)	0 (0.0%)	2 (25.0%)	3 (42.9%)	9 (16.1%)	9 (18.8%)
Mild (%)	4 (12.1%)	13 (43.3%)	1 (6.7%)	4 (36.4%)	5 (62.5%)	3 (42.9%)	10 (17.9%)	20 (41.7%)
Moderate (%)	17 (51.5%)	11 (36.7%)	8 (53.3%)	6 (54.5%)	1 (12.5%)	1 (14.3%)	26 (46.4%)	18 (37.5%)
Severe (%)	6 (18.2%)	0 (0.0%)	5 (33.3%)	1 (9.1%)	0 (0.0%)	0 (0.0%)	11 (19.6%)	1 (2.1%)
Novel comprehensive score								
*N*, Median age (25th-75th)	32, 2.1 (1.6-3.3)	29, 6.4 (5.0-8.6)	15, 1.4 (0.9-3.7)	11, 7.2 (5.6-9.7)	8, 3.5 (0.5-5.1)	7, 10.2 (9.5-13.8)	55, 2.0 (1.1-3.9)	47, 7.9 (5.2-9.7)
Normal (%)	6 (18.8%)	4 (13.8%)	0 (0.0%)	0 (0.0%)	2 (25.0%)	1 (14.3%)	8 (14.5%)	5 (10.6%)
Mild (%)	4 (12.5%)	8 (27.6%)	1 (6.7%)	1 (9.1%)	2 (25.0%)	4 (57.1%)	7 (12.7%)	13 (27.7%)
Moderate (%)	16 (50.0%)	12 (41.4%)	9 (60.0%)	8 (72.7%)	4 (50.0%)	1 (14.3%)	29 (52.7%)	21 (44.7%)
Severe (%)	6 (18.8%)	5 (17.2%)	5 (33.3%)	2 (18.2%)	0 (0.0%)	1 (14.3%)	11 (20.0%)	8 (17.0%)
PNFS								
*N*, Median age (25th-75th)	20, 3.7 (2.0-6.0)	15, 7.7 (5.8-8.4)	10, 2.3 (1.1-6.7)	4, 4.7 (2.6-7.1)	6, 5.5 (3.5-8.2)	3, 10.2 (8.3-12.7)	36, 3.7 (1.8-6.2)	22, 7.1 (5.7-8.7)
Mild (%)	9 (45.0%)	9 (60.0%)	3 (30.0%)	1 (25.0%)	6 (100.0%)	2 (66.7%)	18 (50.0%)	12 (54.5%)
Moderate (%)	3 (15.0%)	2 (13.3%)	1 (10.0%)	1 (25.0%)	0 (0.0%)	1 (33.3%)	4 (11.1%)	4 (18.2%)
Severe (%)	8 (40.0%)	4 (26.7%)	6 (60.0%)	2 (50.0%)	0 (0.0%)	0 (0.0%)	14 (38.9%)	6 (27.3%)
FIB-4								
*N*, Median age (25th-75th)	30, 3.1 (2.1-4.4)	23, 7.6 (5.2-8.7)	15, 1.6 (0.9-5.1)	10, 5.6 (2.6-7.0)	7, 5.5 (3.0-6.7)	4, 11.2 (9.8-13.0)	52, 3.0 (1.8-5.0)	37, 7.5 (5.0-9.4)
Mild (%)	14 (46.7%)	15 (65.2%)	4 (26.7%)	3 (30.0%)	3 (42.9%)	3 (75.0%)	21 (40.4%)	21 (56.8%)
Moderate (%)	7 (23.3%)	3 (13.0%)	7 (46.7%)	3 (30.0%)	4 (57.1%)	1 (25.0%)	18 (34.6%)	7 (18.9%)
Severe (%)	9 (30.0%)	5 (21.7%)	4 (26.7%)	4 (40.0%)	0 (0.0%)	0 (0.0%)	13 (25.0%)	9 (24.3%)
Kulkarni et al^16^								
*N*, Median age (25th-75th)	7, 5.1 (4.6-5.4)	4, 7.4 (5.8-9.6)	4, 2.5 (1.9-5.5)	1, 9.4 (9.4-9.4)	5, 6.0 (3.1-9.0)	2, 9.1 (8.6-9.6)	16, 5.0 (2.8-6.4)	7, 8.7 (7.1-9.8)
No fibrosis (%)	0 (0.0%)	0 (0.0%)	1 (25.0%)	0 (0.0%)	0 (0.0%)	0 (0.0%)	1 (6.3%)	0 (0.0%)
Fibrosis (%)	7 (100.0%)	4 (100.0%)	3 (75.0%)	1 (100.0%)	5 (100.0%)	2 (100.0%)	15 (93.8%)	7 (100.0%)
APRI								
*N*, Median age (25th-75th)	30, 3.1 (2.1-4.4)	27, 6.3 (5.0-8.6)	14, 1.5 (0.9-4.2)	11, 6.3 (2.7-8.2)	7, 4.9 (3.0-6.4)	5, 10.2 (9.0-12.3)	51, 3.0 (1.8-4.9)	43, 7.0 (4.9-9.2)
Mild (%)	30 (100.0%)	27 (100.0%)	14 (100.0%)	11 (100.0%)	7 (100.0%)	5 (100.0%)	51 (100%)	43 (100.0%)
Moderate (%)	0 (0.0%)	0 (0.0%)	0 (0.0%)	0 (0.0%)	0 (0.0%)	0 (0.0%)	0 (0.0%)	0 (0.0%)
Severe (%)	0 (0.0%)	0 (0.0%)	0 (0.0%)	0 (0.0%)	0 (0.0%)	0 (0.0%)	0 (0.0%)	0 (0.0%)
PELD								
*N*, Median age (25th-75th)	23, 3.2 (2.1-5.3)	20, 5.8 (4.0-8.0)	9, 2.0 (1.5-5.7)	4, 4.8 (3.7-5.2)	6, 5.5 (2.7-8.2)	3, 6.5 (5.6-10.9)	38, 3.1 (2.0-5.9)	27, 5.6 (4.3-7.8)
Median score (25th-75th)	-13.5 (-15.3--7.8)	-12.8 (-16.1--11.0)	-11.6 (-14.4--9.7)	-13.6 (-19.8--11.8)	-11.6 (-13.4--9.5)	-12.4 (-12.6--9.9)	-12.9 (-14.9--8.8)	-12.8 (-15.4--10.9)

Established liver disease scoring systems used to classify liver disease included the Pediatric Non-Alcoholic Fatty Liver Disease Score (PNFS), Fibrosis-4 Index (Fib-4), Kulkarni et al,^16^ AST/Platelet Ratio Index (APRI), and the Pediatric End-Stage Liver Disease (PELD) scoring systems. The Novel GSD IX Liver Disease Severity Scoring System included the Novel Lab-Only Score and the Novel Comprehensive Score. All participants were assessed at their baseline and were grouped by their genetic subtype (α2, γ2, or β). Ages are listed in years.

*GSD IX*, glycogen storage disease type IX; *N/A*, not applicable.

### Classification of liver disease in participants with GSD IX using the novel lab-only score

At baseline, the novel lab-only score was able to be calculated for 56 participants. The majority of participants were classified as having either moderate (*n* = 26; 46.4%) or severe (*n* = 11; 19.6%) disease at baseline based on laboratory parameters. At the most recent evaluation, the novel lab-only score was able to be calculated for 48 participants; 20 (41.7%) of participants were classified as having mild disease, 18 (37.5%) as moderate disease, and 1 (2.1%) as having severe disease—a decrease compared with the baseline time point (Table 3).

When classification using the novel lab-only score was compared across the GSD IX subtypes, several trends emerged. In the GSD IX α2 and GSD IX γ2 cohorts at baseline, the majority of pediatric participants were classified as having moderate disease (GSD IX α2: *n* = 17, 51.5%, GSD IX γ2: *n* = 8, 53.3%), with a considerable percentage also being classified as severe (GSD IX α2: *n* = 6, 18.2%, GSD IX γ2: *n* = 5, 33.3%; Table 3). In contrast, most participants in the GSD IX β cohort were classified as having mild disease (*n* = 5, 62.5%). At the most recent time point, both the GSD IX α2 and GSD IX γ2 cohorts demonstrated an increase in the percentage of participants classified as mild or moderate, with a decrease in the percentage of participants being classified as having severe disease (Figure 1, Table 3). The majority of participants with GSD IX β were predicted to have normal or mild disease (*n* = 3; 42.9%, respectively).

### Classification of liver disease in participants with GSD IX using the novel comprehensive score

At baseline, the novel comprehensive score was able to be calculated for 55 participants. Using the comprehensive novel scoring system, most pediatric participants at baseline were classified as having moderate disease (*n* = 29; 52.7%), with a smaller percentage classified as having severe disease (*n* = 11; 20.0%), similar to the novel lab-only score (Table 3). At the most recent time point, the majority of the participants were still classified as moderate (*n* = 21; 44.7%), with smaller percentages classified as having severe disease (*n* = 8; 17.0%) or mild disease (*n* = 13; 27.7%).

Comparing the classification using the novel comprehensive score at baseline across the subtypes, we observed that the majority of participants regardless of subtype were classified as having moderate disease (*n* = 16 [50.0%] GSD IX α2, *n* = 9 [60.0%] GSD IX γ2, and *n* = 4 [50.0%] GSD IX β). Of the remaining participants with GSD IX α2, many were classified as severe (*n* = 6; 18.8%), and the majority of participants with GSD IX γ2 were classified as severe (*n* = 5; 33.3%). Comparing trends across GSD IX subtypes at the most recent evaluation, most participants with GSD IX α2 and γ2 were still classified as moderate (*n* = 12 [41.4%] GSD IX α2, *n* = 8 [72.7%] GSD IX γ2). In the GSD IX α2 cohort, the percentage classified as moderate decreased between baseline and most recent evaluation, with a greater proportion of participants now classified as mild or normal (Figure 1). In contrast, at most recent evaluation, a greater percentage of the GSD IX γ2 cohort was classified as moderate compared with baseline. In the GSD IX β cohort at most recent evaluation, most participants were classified as mild.

### Classification of liver disease in participants with GSD IX using established liver disease scoring systems

#### PNFS

The PNFS could be calculated for 36 participants at baseline visit and 22 participants at the most recent evaluation. Using the severity classification values for the PNFS provided in Table 2, the majority of participants were predicted as having mild disease (*n* = 18, 50.0%) or severe disease (*n* = 14, 38.9%) at baseline, compared with the majority classified as mild disease (*n* = 12, 54.5%) at most recent visit (Table 3). At baseline, the GSD IX γ2 cohort demonstrated the greatest number classified as severe disease (*n* = 6, 60.0%), compared with the majority of participants in the GSD IX β cohort being classified as mild disease (*n* = 6, 100.0%). At the most recent visit, the majority of the GSD IX γ2 and GSD IX β cohorts continued to be predicted as severe and mild disease, respectively (GSD IX γ2: *n* = 2, 50.0%; GSD IX β: *n* = 2, 50.0%). Moreover, the majority of participants in the GSD IX α2 cohort were classified as mild disease (*n* = 9, 45.0%) or severe disease (*n* = 8, 40.0%) at baseline, whereas at most recent visit, the majority of were predicted as mild disease (*n* = 9, 60.0%).

#### FIB-4

The severity classification cutoff values for the FIB-4 are provided in Table 2. Comparing FIB-4 scores at baseline across GSD IX subtypes, the majority of participants with GSD IX α2 were predicted to have mild fibrosis (46.7%), whereas the majority of participants with GSD IX γ2 and GSD IX β were predicted to have moderate fibrosis (13.0% and 57.1%, respectively) (Table 3). At the most recent time point, the majority of participants with GSD IX α2 were again predicted to have mild fibrosis (65.2%), and the majority of participants with GSD IX β were predicted to have mild fibrosis (75.0%). Alternatively, at the most recent time point, the majority of participants with GSD IX γ2 were predicted to have severe fibrosis (40.0%).

#### Proposed methodology by Kulkarni et al

Fibrosis scores by Kulkarni et al^16^ methodology were able to be calculated for 16 participants at baseline visit. Only 1 participant (GSD IX γ2) had no predicted presence of fibrosis, whereas all remaining 15 participants (93.8%) had predicted presence of fibrosis at baseline visit (Table 3). Similarly, Kulkarni et al^16^ methodology was able to be calculated for 7 participants at most recent visit and all participants had predicted presence of fibrosis.

#### APRI

The severity classification cutoff values for the APRI are provided in Table 2. No differences were present between the genotypes at baseline and the most recent time points because no values came close to the 0.7 cutoff value which predicts significant fibrosis.

#### PELD score

The median PELD score at baseline for all participants was −12.9 compared with −12.8 at most recent evaluation (Table 3). None of the median PELD scores in any GSD IX subtype were greater than 0.

### Performance of liver disease scoring systems compared with liver histology severity

With regard to the biopsy specimen using the Ishak and Batts-Ludwig scores, in the GSD IX α2 cohort, 5 of 17 (29.4%) were classified as normal, 4 of 17 (23.5%) as mild, 2 of 17 (11.8%) as moderate, and 6 of 17 (35.3%) as severe. In the GSD IX γ2 cohort, no biopsies were classified as normal, 3 of 11 (27.3%) were classified as mild, 1 of 11 (9.1%) as moderate, and 8 of 11 (63.6%) as severe. Lastly, in the GSD IX β cohort, 1 of 3 (33.3%) was classified as normal and 2 of 3 (66.7%) as mild.

**Figure 2:**
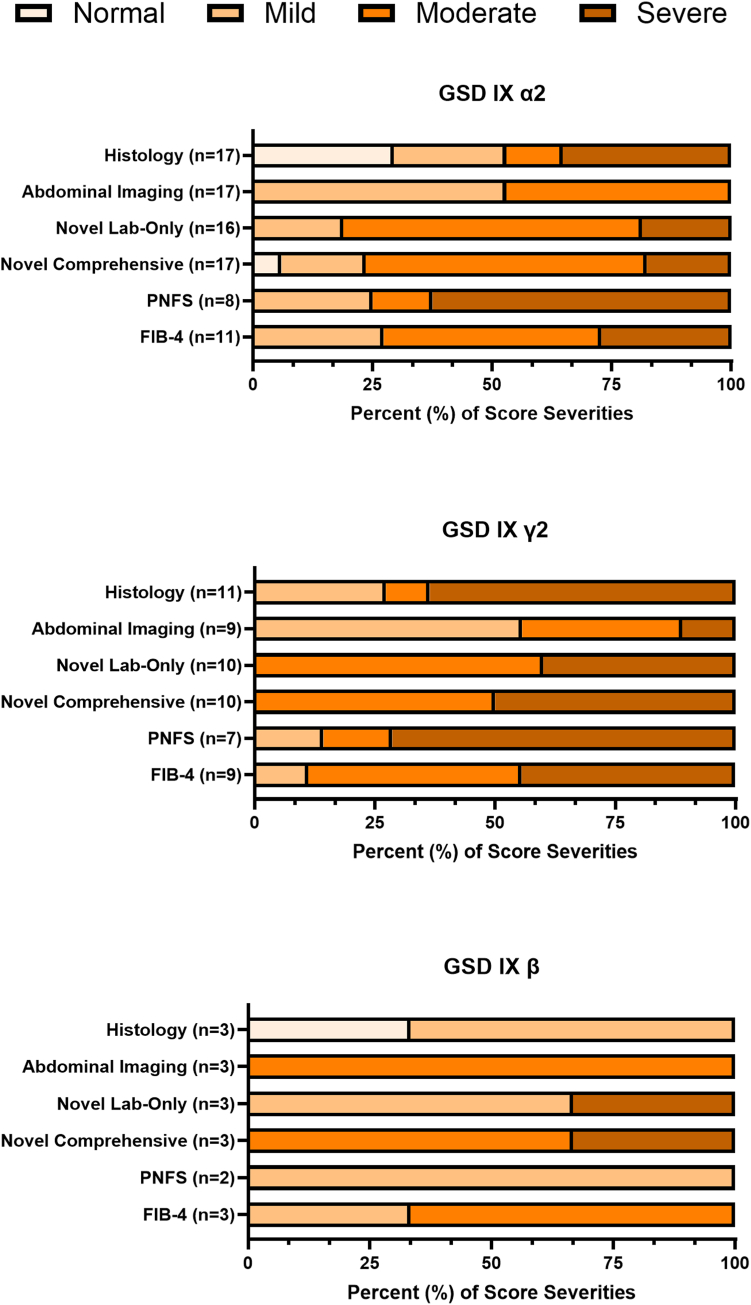
**Liver disease score severity comparisons in pediatric participants with hepatic GSD IX who had a liver biopsy**. The severity classification of participants with biopsy data available at the clinical time point closest to biopsy was separated by the GSD IX subtypes (α2, γ2, and β). The severity was assessed by histology, abdominal imaging, novel lab-only, novel comprehensive, Pediatric Non-Alcoholic Fatty Liver Disease Score (PNFS), and Fibrosis-4 Index (FIB-4) scoring systems. The number of participants included in each score is indicated in parentheses. GSD IX, glycogen storage disease type IX.

Figure 2 provides the severity classification of participants with biopsy available at the clinical time point closest to biopsy for imaging, novel comprehensive scores, and established scores. Using only the participant cohort with liver pathology reports available (*n* = 31), the novel lab-only score correctly classified 8 of 29 (27.6%) of participants, the novel comprehensive score correctly classified 8 of 30 (26.7%) of participants, whereas the PNFS and the FIB-4 correctly classified 8 of 17 (47.1%) and 6 of 23 (26.1%) of participants, respectively. These classification percentages only considered those cases in which the exact category of liver disease severity was correctly predicted (ie, classified as “mild” severity by histology and “mild” severity score by scoring system). Classification rates were not calculated for the Kulkarni methodology or APRI scores given that the Kulkarni method predicted fibrosis with no differentiation between severity, and the APRI classified all participants as having mild disease. When correct classification rates were calculated for the differentiation between mild and severe liver disease (ie, normal or mild vs moderate or severe), the novel lab-only score correctly classified 21 of 29 (72.4%), the novel comprehensive score 20 of 30 (66.7%), the PNFS 14 of 17 (82.4%), and the FIB-4 13 of 23 (56.5%) participants.

## Discussion

Many patients with hepatic GSD IX demonstrate evidence of ongoing and progressive liver disease. To continuously monitor the rate of progression of liver disease, a noninvasive method to grade severity would be of clinical utility. Thus, we proposed a novel liver disease severity scoring system for use in a metabolic hepatic disease population, which uniquely combines key laboratory parameters, abdominal imaging classification, and growth. Furthermore, we assessed the performance of established liver disease severity scores in a hepatic GSD population.

This study compared the well-established and novel scores to predict the liver disease severity, therefore allowing us to explore the performance of each score and the use of imaging alone across the GSD IX subtypes. Using abdominal imaging alone failed to accurately capture the majority of severe disease in the GSD IX α2 and γ2 cohorts. Comparing the lab-only and novel comprehensive scores with the histological classification, we observed that the novel comprehensive score underestimated the severity of disease in the GSD IX α2 and γ2 cohorts, rating substantially more individuals as moderate than scored histologically. Of the established scores, the PNFS seemed to capture liver disease severity most accurately across the GSD IX subtypes. However, both the PNFS and FIB-4 both overestimated the number of participants with mild disease because these scores lack a “normal” value range. The FIB-4 performed similarly to the GSD IX β cohort and seemed to rank the majority of the GSD IX γ2 cohort as moderate, while adequately capturing the severity of the GSD IX α2 cohort.

For calculating the percentage of participants correctly classified by each score (ie, compared with histology severity grade), the PNFS outperformed all novel and established scores. However, using only the percentage of participants classified correctly by category of severity, none of the scores demonstrated clinically useful accuracy; rather, the accuracy was greatly improved when classification was grouped by normal and mild versus moderate and severe disease. These findings suggest that these scores may have clinical utility in certain settings, such as identifying participants concerning for severe disease, and may be able to guide decision making for determining the need for more invasive monitoring, such as repeat liver biopsy. Unexpectedly, the novel lab-only score outperformed the novel comprehensive score (72.4% vs 66.7% accuracy). However, these scoring systems measure different hepatic findings, with imaging being primarily utilized for the detection of fibrosis or cirrhosis (novel comprehensive score), whereas laboratory parameters may best be suited to reflect the degree of liver damage (novel lab-only score). Future investigations should consider the utility of other noninvasive liver imaging techniques, such as elastography, as well as biochemical indicators of liver fibrosis, such as the FibroTest, the Enhanced Liver Fibrosis test, and fibroblast growth factor 21 (FGF21), in hepatic GSD IX to assess their ability to predict disease severity.

Interestingly, when we compared the laboratory parameters used for calculation of each score, we can see that the PELD score relies on synthetic biomarkers (ie, bilirubin, INR, and albumin).^7^^,^^28^ The overall low PELD scores in our cohort were unsurprising because the PELD score is often modified for metabolic participants, including GSD IX, using an “exception” because this score fails to truly capture the severity of disease because of the lack of synthetic dysfunction often observed in this population.^32^ As such, the PELD score is not suited for use in metabolic disorders. Similarly, the APRI is a ratio of the degree of elevation of AST above the upper limit of normal divided by the platelet count.^30^ As reported in previous studies, participants with GSD IX are similar to other hepatic GSDs (such as GSD III and VI) in which there is liver disease progression; yet, patients do not often experience derangements in synthetic function, including thrombocytopenia.^22^^,^^33^^,^^34^ This supports the lack of APRI elevation in our cohort and reinforces why the APRI score was of limited utility for differentiation of liver disease severity in GSD IX.

The primary limitations of our study stem from the retrospective nature of data collection. Calculations of “correct” classification rates were limited by the smaller cohort for which liver biopsy was available. Therefore, observing the performance of these scores in an expanded population of participants with hepatic GSD IX may provide further insight. In addition, the distribution of participants was skewed toward the GSD IX α2 subtype because of the greater frequency of the subtype and X-linked inheritance pattern.^7^ Interpretation of the values for the established adult scoring systems (FIB-4 and APRI) should be viewed with caution because these scores are not validated for use in a pediatric population; the cutoff values used to classify severity were based on adult values, which may not be accurate in a pediatric population. Our incorporation of growth parameters into the novel score was limited by the lack of midparental height data for our participant population, especially considering the diversity of ethnic backgrounds represented. Catch-up growth is common in hepatic GSD IX when under good metabolic control; therefore, the incorporation of growth as a parameter for assessing liver disease beyond childhood is unclear. Given the limited number of females in our study (21.1%; Supplemental Table 2), we were unable to draw conclusions about the impact of sex on liver disease scores, and additional studies assessing the impact of sex on liver disease in GSD IX are warranted. Lastly, we assessed the severity of liver disease at baseline and most recent visit; future investigations should explore liver disease scores in individual participants over time and consider assessing the specificity and sensitivity of different scoring systems.

The novel GSD IX liver disease severity scores predicted that a significant portion of individuals with GSD IX had moderate to severe liver fibrosis at baseline and most recent evaluations, a finding also reflected by the median of the established severity scores. Of the established liver disease scores, the PNFS demonstrated the most reliable performance for differentiation between mild and severe liver disease and could be a useful scoring system for use in a metabolic disease population, such as GSD IX. The PNFS relies on ALT, alkaline phosphatase, platelet count, and GGT, whereas, the proposed novel lab-only score utilizes ALT, bilirubin, and GGT. The PNFS demonstrated utility for clinical practice as a noninvasive laboratory parameter-based clinical score for identifying the participants who may require additional invasive procedures, such as repeat liver biopsy to monitor disease severity. Use of the PNFS may be especially beneficial for monitoring in resource-limited areas. The accuracy of liver disease prediction scores was greatly increased when comparing rates of differentiating between the extremes (mild and severe disease), a finding previously reported with the APRI in determining severity of NAFLD.^21^ Our data suggest that prediction scores may provide a useful clinical tool to noninvasively gauge severity of liver disease—particularly when differentiating between mild and severe disease—in GSD IX and other hepatic GSDs, which all require longitudinal monitoring. Future studies with modifications or further refinement will verify these scoring systems within larger cohorts and animal models of hepatic GSD IX. At this time, utility of liver biopsy should not be underestimated, especially because many patients retain stable synthetic function.

Despite the varying severity of the hepatic GSD IX, a noninvasive method of monitoring liver disease progression in this population is unavailable, and liver biopsy remains the gold standard. We proposed 2 novel hepatic GSD IX-specific liver disease severity scoring systems and compared their performance with that of established scoring systems traditionally used for other liver diseases. All scores were compared with that of the gold-standard liver biopsy fibrosis stage, and we found that the PNFS demonstrated the highest accuracy in liver disease severity classification. Although the examined scoring systems demonstrated the greatest severity of liver disease among participants with GSD IX γ2, several participants with GSD IX α2 also demonstrated severe disease. Liver disease severity prediction scores can provide a useful clinical tool in noninvasively gauging severity of liver disease in GSD IX, and additional work is needed to determine the utility of these measurement tools in clinical trials for the treatment of hepatic GSDs. Regardless, liver biopsy remains an important part of clinical care in these patients and should be performed where indicated in close collaboration with the hepatology team. Further work is needed to refine accurate noninvasive metabolic clinical liver disease severity scores for all hepatic GSDs.

## Data Availability

Requests to access data should be directed to the corresponding author.

## ORCID

Priya S. Kishnani: https://orcid.org/0000-0001-8251-909X

## Conflict of Interest

Priya S. Kishnani, Rebecca L. Koch, and William R. Jeck have received research/grant support from Kriya Therapeutics. Priya S. Kishnani has equity and received consulting fees and honoraria from Kriya Therapeutics. All other authors declare no conflicts of interest.
