# Supplementary material for: Evaluating and monitoring liver disease severity in glycogen storage disease type IX: Performance of novel and established clinical scores
Source: Genet Med Open. 2025 Sep 10;4:103455. doi: 10.1016/j.gimo.2025.103455 (PMC13196317; doi:10.1016/j.gimo.2025.103455)
Supplement: Supplementary Material [file mmc1.docx]

# Supplementary Table 1. Division of Batts-Ludwig and Ishak pathology scores into severity classifications for comparison to scoring systems.

The Batts-Ludwig and Ishak fibrosis scoring systems were classified by fibrosis severity: normal (no fibrosis), mild, moderate, or severe/cirrhosis by grades to allow for easier comparison of correct classification rates to the proposed novel and established scoring systems.

| Liver Disease (Fibrosis) Severity | Batts-Ludwig | Ishak |
| --- | --- | --- |
| Normal | Grade 0 | Grade 0 |
| Mild | Grade 1 | Grade 1-2 |
| Moderate | Grade 2 | Grade 3 |
| Severe / Cirrhosis | Grade 3 | Grade 4 |
|  | Grade 4 | Grade 5-6 |

# Supplementary Table 2. Demographic characteristics for the pediatric participants with hepatic glycogen storage disease type IX (GSD IX).

International pediatric participants with a confirmed diagnosis of GSD IX were included. GSD IX subtype was determined through genetic analysis by the presence of pathogenic variant(s) in the phosphorylase kinase (PhK) genes (*PHKA2* [α2], *PHKG2* [γ2], and *PHKB* [β]). Additional demographic characteristics, including sex, race, and ethnicity, are indicated.

|  | **Pediatric Population (N=57)** |
| --- | --- |
| **GSD IX Subtype** | |
| GSD IX α2 | 34 (59.6%) |
| GSD IX γ2 | 15 (26.3%) |
| GSD IX β | 8 (14.0%) |
| **Gender** | |
| Female | 12 (21.1%) |
| Male | 45 (78.9%) |
| **Race** | |
| White | 36 (63.2%) |
| Black or African American | 2 (3.5%) |
| Asian | 7 (12.3%) |
| American Indian or Alaska Native | 0 (0.0%) |
| Native Hawaiian or Pacific Islander | 0 (0.0%) |
| Other | 8 (14.0%) |
| Unknown | 4 (7.0%) |
| **Ethnicity** | |
| Hispanic/Latino | 5 (8.8%) |
| Not Hispanic/Latino | 37 (64.9%) |
| Unknown | 15 (26.3%) |

#

# Supplementary Table 3. Hepatic glycogen storage disease type IX (GSD IX) patient genetic variants.

Pediatric participants with a genetically-confirmed diagnosis of hepatic GSD IX were included in the study. Patients are grouped by affected gene (*PHKA2* [NM_000292.3], *PHKG2* [NM_000294.3], or *PHKB* [NM_000293.3]). *PHKA2* is located on the X chromosome and the listed variant is hemizygous with the exceptions of *^h^* which indicates females who are heterozygous and have a clinical diagnosis of GSD IX α2. A variant’s predicted effect and location on the gene was identified using VarSome (Version 11.2, accessed January 2022). ^a^ represents patients previously reported^1,2^, ^b-g^ represent familial relationships, respectively, and *^i^*participant 42 is homozygous for a deletion of exon 6 in *PHKB*.

| **ID** | **Gene** | **Variant 1** | | | | **Variant 2** | | | |
| --- | --- | --- | --- | --- | --- | --- | --- | --- | --- |
|  |  | **Variant Description** | **Predicted Effect** | **Location** | **ACMG/AMP Classification** | **Variant Description** | **Predicted Effect** | **Location** | **ACMG/AMP Classification** |
| **2*^a^*** | *PHKA2* | c.3614C>T  p.(Pro1205Leu)  NC_000023.11:g.18893579G>A | Missense | Exon 33 | Pathogenic (PS4, PM2_Supporting, PP1, PP3, PP4_Strong) |  |  |  |  |
| **6*^a^*** | *PHKA2* | c.1576G>A  p.(Asp526Asn)  NC_000023.11:g.18924519C>T | Missense | Exon 16 | Variant of uncertain significance (BS4, PP3) |  |  |  |  |
| **7*^a^*** | *PHKA2* | c.112G>C  p.(Glu38Gln)  NC_000023.11:g.18954379C>G | Missense | Exon 2 | Likely benign (BS1, BP5) | c.811del  p.(Glu271LysfsTer3)  NC_000023.11:g.18941583del | Frameshift | Exon 8 | Likely pathogenic (PVS1, PM2_Supporting) |
| **8*^a^*** | *PHKA2* | c.884G>A  p.(Arg295His)  NC_000023.11:g.18940029C>T | Missense | Exon 9 | Likely pathogenic (PM1, PM2_Supporting, PP3, PP4_Moderate) |  |  |  |  |
| **10*^a^*** | *PHKA2* | c.883C>T  p.(Arg295Cys)  NC_000023.11:g.18940030G>A | Missense | Exon 9 | Likely pathogenic (PM1, PM2_Supporting, PP1, PP3, PP4_Moderate) |  |  |  |  |
| **11*^a^*** | *PHKA2* | c.893G>C  p.(Arg298Pro)  NC_000023.11:g.18940020C>G | Missense | Exon 9 | Variant of uncertain significance (PM2_Supporting, PP3, PP4_Moderate) |  |  |  |  |
| **13*^a^*** | *PHKA2* | c.3334G>T  p.(Glu1112Ter)  NC_000023.11:g.18895140C>A | Nonsense | Exon 31 | Pathogenic (PVS1, PM2_Supporting, PP4_Moderate) |  |  |  |  |
| **15*^a^*** | *PHKA2* | c.133C>T  p.(Arg45Trp)  NC_000023.11:g.18954358G>A | Missense | Exon 2 | Likely pathogenic (PM1, PM2_Supporting, PP3, PP4_Moderate) |  |  |  |  |
| **16*^a^*** | *PHKA2* | c.1715-2A>G  p.?  NC_000023.11:g.18924136T>C | Splice site | Intron 16 | Likely pathogenic (PVS1_Strong, PM2_Supporting, PP4_Moderate) |  |  |  |  |
| **17** | *PHKA2* | c.3505C>T  p.(Gln1169Ter)  NC_000023.11:g.18894236G>A | Nonsense | Exon 32 | Likely pathogenic (PVS1_Strong, PM2_Supporting, PP4_Moderate) |  |  |  |  |
| **18*^a^*** | *PHKA2* | c.883C>T  p.(Arg295Cys)  NC_000023.11:g.18940030G>A | Missense | Exon 9 | Likely pathogenic (PM1, PM2_Supporting, PP1, PP3, PP4_Moderate) |  |  |  |  |
| **21*^a^*** | *PHKA2* | c.1963+3A>G*^h^*  p.?  NC_000023.11:g.18920029T>C | Splice site | Intron 18 | Variant of uncertain significance (PM2_Supporting, PP3, PP4_Moderate) |  |  |  |  |
| **22*^a^*** | *PHKA2* | c.133C>T  p.(Arg45Trp)  NC_000023.11:g.18954358G>A | Missense | Exon 2 | Likely pathogenic (PM1, PM2_Supporting, PP3, PP4_Moderate) |  |  |  |  |
| **23*^a^*** | *PHKA2* | c.133C>T  p.(Arg45Trp)  NC_000023.11:g.18954358G>A | Missense | Exon 2 | Likely pathogenic (PM1, PM2_Supporting, PP3, PP4_Moderate) |  |  |  |  |
| **27*^a^*** | *PHKA2* | c.749C>T  p.(Ser250Leu)  NC_000023.11:g.18941644G>A | Missense | Exon 8 | Variant of uncertain significance (BP4) |  |  |  |  |
| **31*^a^*** | *PHKA2* | c.883C>T  p.(Arg295Cys)  NC_000023.11:g.18940030G>A | Missense | Exon 9 | Likely pathogenic (PM1, PM2_Supporting, PP1, PP3, PP4_Moderate) |  |  |  |  |
| **32*^b^*** | *PHKA2* | c.893G>C  p.(Arg298Pro)  NC_000023.11:g.18940020C>G | Missense | Exon 9 | Variant of uncertain significance (PM2_Supporting, PP3, PP4_Moderate) |  |  |  |  |
| **33*^b^*** | *PHKA2* | c.893G>C  p.(Arg298Pro)  NC_000023.11:g.18940020C>G | Missense | Exon 9 | Variant of uncertain significance (PM2_Supporting, PP3, PP4_Moderate) |  |  |  |  |
| **44** | *PHKA2* | c.3614C>T  p.(Pro1205Leu)  NC_000023.11:g.18893579G>A | Missense | Exon 33 | Pathogenic (PS4, PM2_Supporting, PP1, PP3, PP4_Strong) |  |  |  |  |
| **45** | *PHKA2* | c.883C>T  p.(Arg295Cys)  NC_000023.11:g.18940030G>A | Missense | Exon 9 | Likely pathogenic (PM1, PM2_Supporting, PP1, PP3, PP4_Moderate) |  |  |  |  |
| **47** | *PHKA2* | c.3334G>A  p.(Glu1112Lys)  NC_000023.11:g.18895140C>T | Missense | Exon 31 | Variant of uncertain significance (PM2_Supporting, PP1, PP3, PP4_Moderate) |  |  |  |  |
| **48** | *PHKA2* | c.133C>T  p.(Arg45Trp)  NC_000023.11:g.18954358G>A | Missense | Exon 2 | Likely pathogenic (PM1, PM2_Supporting, PP3, PP4_Moderate) |  |  |  |  |
| **49** | *PHKA2* | c.2785G>C  p.(Ala929Pro)  NC_000023.11:g.18906516C>G | Missense | Exon 25 | Variant of uncertain significance (PM2_Supporting, PP3) | c.2735T>C  p.(Met912Thr)  NC_000023.11:g.18906566A>G | Missense | Exon 25 | Variant of uncertain significance (PM2_Supporting, PP3) |
| **50** | *PHKA2* | c.884G>A  p.(Arg295His)  NC_000023.11:g.18940029C>T | Missense | Exon 9 | Likely pathogenic (PM1, PM2_Supporting, PP3, PP4_Moderate) |  |  |  |  |
| **51** | *PHKA2* | c.262del  p.(Leu88PhefsTer5)*^h^*  NC_000023.11:g.18952517del | Frameshift | Exon 3 | Likely pathogenic (PVS1, PM2_Supporting) |  |  |  |  |
| **53*^c^*** | *PHKA2* | c.1482G>C  p.(Leu494Phe)  NC_000023.11:g.18925755C>G | Missense | Exon 15 | Variant of uncertain significance (PM2_Supporting, PP3) |  |  |  |  |
| **54*^c^*** | *PHKA2* | c.1482G>C  p.(Leu494Phe)  NC_000023.11:g.18925755C>G | Missense | Exon 15 | Variant of uncertain significance (PM2_Supporting, PP3) |  |  |  |  |
| **56*^d^*** | *PHKA2* | c.3586_3621del  p.(Ile1196_Gly1207del)  NC_000023.11:g.18893572_18893607del | Deletion | Exon 33 | Variant of uncertain significance (PM2_Supporting, PM4) |  |  |  |  |
| **57** | *PHKA2* | c.1775G>T  p.(Gly592Val)  NC_000023.11:g.18924074C>A | Missense | Exon 17 | Variant of uncertain significance (PM2_Supporting, PP3) |  |  |  |  |
| **59*^d^*** | *PHKA2* | c.3586_3621del  p.(Ile1196_Gly1207del)*^h^*  NC_000023.11:g.18893572_18893607del | Deletion | Exon 33 | Variant of uncertain significance (PM2_Supporting, PM4) |  |  |  |  |
| **62** | *PHKA2* | c.4C>G  p.(Arg2Gly)  NC_000023.11:g.18983929G>C | Missense | Exon 1 | Variant of uncertain significance (PP1, PP4) |  |  |  |  |
| **65** | *PHKA2* | c.883C>T  p.(Arg295Cys)  NC_000023.11:g.18940030G>A | Missense | Exon 9 | Likely pathogenic (PM1, PM2_Supporting, PP1, PP3, PP4_Moderate) |  |  |  |  |
| **71** | *PHKA2* | c.134G>A  p.(Arg45Gln)  NC_000023.11:g.18954357C>T | Missense | Exon 2 | Likely pathogenic (PM1, PM2_Supporting, PP3, PP4_Moderate) |  |  |  |  |
| **72** | *PHKA2* | c.3614C>T  p.(Pro1205Leu)  NC_000023.11:g.18893579G>A | Missense | Exon 33 | Pathogenic (PS4, PM2_Supporting, PP1, PP3, PP4_Strong) |  |  |  |  |
| **9** | *PHKG2* | c.900G>A  p.(Trp300Ter)  NC_000016.10:g.30756688G>A | Nonsense | Exon 9 | Likely pathogenic (PVS1_Strong, PM2_Supporting, PP4_Moderate) | c.1073A>G  p.(Tyr358Cys)  NC_000016.10:g.30756949A>G | Missense | Exon 10 | Variant of uncertain significance (PM2_Supporting, PP3, PP4_Moderate) |
| **12*^ae^*** | *PHKG2* | c.96-11G>A  p.?  NC_000016.10:g.30751095G>A | Non-coding variant | Intron 2 | Likely pathogenic (PS3_Moderate, PM2_Supporting, PM3, PP3, PP4_Moderate) | c.556+1069T>G  p.?  NC_000016.10:g.30754626T>G | Intronic pseudoexon | Intron 6 | Likely pathogenic (PS3_Moderate, PM2_Supporting, PM3_Supporting, PP3, PP4_Moderate) |
| **14*^a^*** | *PHKG2* | c.247C>T  p.(Gln83Ter)  NC_000016.10:g.30751257C>T | Nonsense | Exon 3 | Pathogenic (PVS1, PM2_Supporting, PP4_Moderate) | c.96-11G>A  p.?  NC_000016.10:g.30751095G>A | Non-coding | Intron 2 | Likely pathogenic (PS3_Moderate, PM2_Supporting, PM3, PP3, PP4_Moderate) |
| **19*^e^*** | *PHKG2* | c.96-11G>A  p.?  NC_000016.10:g.30751095G>A | Non-coding | Intron 2 | Likely pathogenic (PS3_Moderate, PM2_Supporting, PM3, PP3, PP4_Moderate) | c.556+1069T>G  p.?  NC_000016.10:g.30754626T>G | Intronic pseudoexon | Intron 6 | Likely pathogenic (PS3_Moderate, PM2_Supporting, PM3_Supporting, PP3, PP4_Moderate) |
| **36** | *PHKG2* | c.698T>C  p.(Phe233Ser)  NC_000016.10:g.30756417T>C | Missense | Exon 8 | Likely pathogenic (PS3_Supporting, PM1, PM2_Supporting, PP3, PP4_Moderate) | c.698T>C  p.(Phe233Ser)  NC_000016.10:g.30756417T>C | Missense | Exon 8 | Likely pathogenic (PS3_Supporting, PM1, PM2_Supporting, PP3, PP4_Moderate) |
| **37** | *PHKG2* | c.237_238del  p.(Ile80ProfsTer21)  NC_000016.10:g.30751247_30751248del | Frameshift | Exon 3 | Likely pathogenic (PVS1, PM2_Supporting) | c.517G>A  p.(Gly173Arg)  NC_000016.10:g.30753518G>A | Missense | Exon 6 | Variant of uncertain significance (PM2_Supporting, PP3) |
| **38** | *PHKG2* | c.925C>T  p.(Arg309Trp)  NC_000016.10:g.30756713C>T | Missense | Exon 9 | Variant of uncertain significance (PM2_Supporting, PM3_Supporting, PP3, PP4_Moderate) | c.925C>T  p.(Arg309Trp)  NC_000016.10:g.30756713C>T | Missense | Exon 9 | Variant of uncertain significance (PM2_Supporting, PM3_Supporting, PP3, PP4_Moderate) |
| **39** | *PHKG2* | c.863A>G  p.(His288Arg)  NC_000016.10:g.30756651A>G | Missense | Exon 9 | Variant of uncertain significance (PM2_Supporting, PP3) | c.863A>G  p.(His288Arg)  NC_000016.10:g.30756651A>G | Missense | Exon 9 | Variant of uncertain significance (PM2_Supporting, PP3) |
| **40*^f^*** | *PHKG2* | c.383del  p.(Lys128ArgfsTer14)  NC_000016.10:g.30753288del | Frameshift | Exon 5 | Likely pathogenic (PVS1, PM2_Supporting) | c.383del  p.(Lys128ArgfsTer14)  NC_000016.10:g.30753288del | Frameshift | Exon 5 | Likely pathogenic (PVS1, PM2_Supporting) |
| **41*^f^*** | *PHKG2* | c.383del;  p.(Lys128ArgfsTer14)  NC_000016.10:g.30753288del | Frameshift | Exon 5 | Likely pathogenic (PVS1, PM2_Supporting) | c.383del  p.(Lys128ArgfsTer14)  NC_000016.10:g.30753288del | Frameshift | Exon 5 | Likely pathogenic (PVS1, PM2_Supporting) |
| **43** | *PHKG2* | c.698T>C  p.(Phe233Ser)  NC_000016.10:g.30756417T>C | Missense | Exon 8 | Likely pathogenic (PS3_Supporting, PM1, PM2_Supporting, PP3, PP4_Moderate) | c.698T>C  p.(Phe233Ser)  NC_000016.10:g.30756417T>C | Missense | Exon 8 | Likely pathogenic (PS3_Supporting, PM1, PM2_Supporting, PP3, PP4_Moderate) |
| **52** | *PHKG2* | c.647+5G>A  p.?  NC_000016.10:g.30756277G>A | Splice site | Intron 7 | Variant of uncertain significance (PM2_Supporting, PP3) | c.647+5G>A  p.?  NC_000016.10:g.30756277G>A | Splice site | Intron 7 | Variant of uncertain significance (PM2_Supporting, PP3) |
| **64** | *PHKG2* | c.22G>T  p.(Glu8Ter)  NC_000016.10:g.30748842G>T | Nonsense | Exon 2 | Pathogenic (PVS1, PM2_Supporting, PP4_Moderate) | c.835C>T  p.(Arg279Cys)  NC_000016.10:g.30756623C>T | Missense | Exon 9 | Variant of uncertain significance (PM2_Supporting, PM3_Supporting) |
| **74** | *PHKG2* | c.835C>T  p.(Arg279Cys)  NC_000016.10:g.30756623C>T | Missense | Exon 9 | Variant of uncertain significance (PM2_Supporting, PM3_Supporting) | c.835C>T  p.(Arg279Cys)  NC_000016.10:g.30756623C>T | Missense | Exon 9 | Variant of uncertain significance (PM2_Supporting, PM3_Supporting) |
| **75** | *PHKG2* | c.835C>T  p.(Arg279Cys)  NC_000016.10:g.30756623C>T | Missense | Exon 9 | Variant of uncertain significance (PM2_Supporting, PM3_Supporting) | c.835C>T  p.(Arg279Cys) NC_000016.10:g.30756623C>T | Missense | Exon 9 | Variant of uncertain significance (PM2_Supporting, PM3_Supporting) |
| **20** | *PHKB* | c.1969C>T  p.(Gln657Ter)  NC_000016.10:g.47650919C>T | Nonsense | Exon 20 | Pathogenic (PVS1, PM2_Supporting, PP4_Moderate) | c.707C>A  p.(Ser236Ter)  NC_000016.10:g.47547545C>A | Nonsense | Exon 7 | Pathogenic (PVS1, PM2_Supporting, PM3_Supporting) |
| **26*^a^*** | *PHKB* | c.1257T>A  p.(Tyr419Ter)  NC_000016.10:g.47596425T>A | Nonsense | Exon 14 | Pathogenic (PVS1, PM2_Supporting, PP4_Moderate) | c.2336+4del  p.?  NC_000016.10:g.47663738del | Splice site | Intron 25 | Variant of uncertain significance (PM2_Supporting, PM3_Supporting, PP3) |
| **29*^g^*** | *PHKB* | c.1090G>T  p.(Glu364Ter)  NC_000016.10:g.47593521G>T | Nonsense | Exon 11 | Pathogenic (PVS1, PM2_Supporting, PP4) | c.3124del  p.(Leu1042Ter)  NC_000016.10:g.47698568del | Nonsense | Exon 31 | Likely pathogenic (PVS1_Strong, PM2_Supporting, PM3_Supporting, PP4) |
| **30*^g^*** | *PHKB* | c.1090G>T  p.(Glu364Ter)  NC_000016.10:g.47593521G>T | Nonsense | Exon 11 | Pathogenic (PVS1, PM2_Supporting, PP4) | c.3124del  p.(Leu1042Ter)  NC_000016.10:g.47698568del | Nonsense | Exon 31 | Likely pathogenic (PVS1_Strong, PM2_Supporting, PM3_Supporting, PP4) |
| **42*^i^*** | *PHKB* | c.(513+1_514-1)_(594+1_595-1)del;  p.?  NC_000016.10:g.(47511773_47515520)_(47515602_47547432)del | Deletion | Exon 6 | Variant of uncertain significance (PVS1_Moderate, PM2_Supporting) | c.(513+1_514-1)_(594+1_595-1)del;  p.?  NC_000016.10:g.(47511773_47515520)_(47515602_47547432)del | Deletion | Exon 6 | Variant of uncertain significance (PVS1_Moderate, PM2_Supporting) |
| **46** | *PHKB* | c.1688C>A  p.(Ser563Ter)  NC_000016.10:g.47648612C>A | Nonsense | Exon 18 | Pathogenic (PVS1, PM2_Supporting, PP4) | c.1688C>A  p.(Ser563Ter) NC_000016.10:g.47648612C>A | Nonsense | Exon 18 | Pathogenic (PVS1, PM2_Supporting, PP4) |
| **69** | *PHKB* | c.1972-2A>G  p.?  NC_000016.10:g.47660504A>G | Splice site | Intron 20 | Likely pathogenic (PVS1, PM2_Supporting) | c.2181del  p.(Leu728PhefsTer4)  NC_000016.10:g.47660804del | Frameshift | Exon 22 | Likely pathogenic (PVS1, PM2_Supporting) |
| **73** | *PHKB* | c.573_577del  p.(Gln191HisfsTer5)  NC_000016.10:g.47515580_47515584del | Frameshift | Exon 7 | Likely pathogenic (PVS1, PM2_Supporting) | c.1972-2A>G  p.?  NC_000016.10:g.47660504A>G | Splice site | Intron 20 | Likely pathogenic (PVS1, PM2_Supporting) |

**References:**

1. Bali DS, Goldstein JL, Fredrickson K, et al. Clinical and Molecular Variability in Patients with PHKA2 Variants and Liver Phosphorylase b Kinase Deficiency. JIMD Rep*.* 2017;37:63-72.

2. Bali DS, Goldstein JL, Fredrickson K, et al. Variability of disease spectrum in children with liver phosphorylase kinase deficiency caused by mutations in the PHKG2 gene. Mol Genet Metab*.* 2014;111(3):309-313.
